# Synthetic tetracycline-inducible regulatory networks: computer-aided design of dynamic phenotypes

**DOI:** 10.1186/1752-0509-1-7

**Published:** 2007-01-09

**Authors:** Vassilios Sotiropoulos, Yiannis N Kaznessis

**Affiliations:** 1 Department of Chemical Engineering and Materials Science, and Digital Technology Center, University of Minnesota, 421 Washington Ave SE, Minneapolis, MN 55455 USA

## Abstract

**Background:**

Tightly regulated gene networks, precisely controlling the expression of protein molecules, have received considerable interest by the biomedical community due to their promising applications. Among the most well studied inducible transcription systems are the tetracycline regulatory expression systems based on the tetracycline resistance operon of Escherichia coli, Tet-Off (tTA) and Tet-On (rtTA). Despite their initial success and improved designs, limitations still persist, such as low inducer sensitivity. Instead of looking at these networks statically, and simply changing or mutating the promoter and operator regions with trial and error, a systematic investigation of the dynamic behavior of the network can result in rational design of regulatory gene expression systems. Sophisticated algorithms can accurately capture the dynamical behavior of gene networks. With computer aided design, we aim to improve the synthesis of regulatory networks and propose new designs that enable tighter control of expression.

**Results:**

In this paper we engineer novel networks by recombining existing genes or part of genes. We synthesize four novel regulatory networks based on the Tet-Off and Tet-On systems. We model all the known individual biomolecular interactions involved in transcription, translation, regulation and induction. With multiple time-scale stochastic-discrete and stochastic-continuous models we accurately capture the transient and steady state dynamics of these networks. Important biomolecular interactions are identified and the strength of the interactions engineered to satisfy design criteria. A set of clear design rules is developed and appropriate mutants of regulatory proteins and operator sites are proposed.

**Conclusion:**

The complexity of biomolecular interactions is accurately captured through computer simulations. Computer simulations allow us to look into the molecular level, portray the dynamic behavior of gene regulatory networks and rationally engineer novel ones with useful applications. We are able to propose, test and accept or reject design principles for each network. Guided by simulations, we develop a set of design principles for novel tetracycline-inducible networks.

## Background

Tightly regulated gene networks, precisely controlling the expression of protein molecules, have received considerable interest [1] by the biomedical community due to their promising applications. Recently, regulatory gene networks have been used in exciting biomedical applications, such as delivery of therapeutic genes, treating cancer, diabetes, and other diseases [2,3]. Proposed designs have been tested both *in vitro *and *in vivo *[2,3], leading to encouraging results.

Desirable characteristics of a fine tuned system include silent expression in the absence of inducer (low expression leakiness), high induced expression, high specificity and sensitivity to inducers, quick response to inducers, regulation by an orally bioavailable inducer, minimal or no immune impact to the host and finally *in vivo *applicability. The most widely used inducible transcription systems that largely meet these criteria are the tetracycline regulatory expression systems based on the tetracycline resistance operon of *Escherichia coli *(*E. coli*) [4]. Tet-Off and Tet-On systems, also known as rTA and rtTA, respectively, are among the most well studied systems of this category [5-8].

Tet -Off, first employed by Gossen and Bujard [7] is a binary transgenic system in which expression of a target transgene is dependent on the activity of an inducible transcriptional activator. The transcriptional activator is a tetracycline-controlled transactivator protein (tTA), which is a fusion between the Tet repressor DNA binding protein (TetR) and a transactivator, such as VP16 of the herpes simplex virus. The target gene is under transcriptional control of a tetracycline-responsive promoter element (TRE), a seven *Tet operators *(*TetO*) moiety placed upstream of a minimal promoter, typically derived from the human cytomegalovirus (hCMV). Expression of the transgene can be regulated both reversibly (expression is turned back on again when tetracycline has cleared out of the system) and quantitatively by exposing the system to varying concentrations of tetracycline (Tc), or Tc derivatives such as doxycycline (Dox) or minocycline. Transcription is silenced when tetracycline derivatives are administered, since TetR loses its affinity for *TetO*.

While Tet-Off requires the absence of Tc for expression of the transgene, in the Tet-On system the transgene is expressed when Tc or its analogues are present. Four amino acid substitutions on the TetR sequence led to reverse TetR, which binds *TetO *sequences only in the presence of Tc. Reverse TetR fused with a transactivator domain (rtTA) has the reverse phenotype of tTA, allowing transgene expression in the presence of Tc or its analogues. This last characteristic makes Tet-On systems more attractive than Tet-Off, since in general, organisms are more easily saturated with an inducer than depleted of it.

Despite their initial success, both systems (tTA and rtTA) still face limitations that need to be addressed before routinely using them in human gene therapies:

• High-level expression of Tet-OFF or Tet-ON transactivators might cause cellular toxicity, or selective pressure against the stable incorporation of vectors expressing the transactivators.

• Therapeutic gene expression leakage is still present despite the strength of biomolecular interactions comprising Tet-OFF or Tet-ON. For example, there is residual affinity of Tet-ON for *TetO*, even in the absence of Tc.

• Only traces of Tc or Dox appear to be sufficient for silencing expression in Tet-OFF, requiring days before the system's behavior is reversed.

• Fairly high levels of Dox are required for Tet-ON to be activated, a concentration that cannot be readily achieved in the brain of mice.

To address these issues, novel tetracycline regulated systems were engineered that display both low basal expression levels and higher affinity for Dox [9,10]. Additionally acidic activation domains can replace VP16, creating a wide selection of possible transactivators. Another strategy employed to reduce basal activity was the fusion of TetR with a KRAB domain (tTS) [11], which led to repression of unwanted transgene when Tc was absent without affecting expression in the presence of Tc. Combined tTS and rtTA [12] systems demonstrate promising results. Moreover autoregulated expression vectors have been successfully used to control expression of Tet-OFF or Tet-ON [13]. Additional strategies working towards improving the original Tet designs include the use of adenovirus vector systems [14] and the use of histone deacetylases in mammalian cells [15].

Nonetheless, limitations persist. Instead of looking at these networks statically, and simply changing or mutating the promoter and operator regions with trial and error, a systematic investigation of the dynamic behavior of the network can result in rational design of regulatory gene expression systems. The observation that gene networks are inherently stochastic [16], allow to numerically simulate complex networks of regulated biological reactions. By combining fast supercomputers and greater knowledge of the molecular mechanisms of gene expression, we can numerically simulate the stochastic dynamics of gene networks and understand in depth how components of the network affect system level performance. Multiple time-scale algorithms[17] are able to accurate capture the dynamical behavior of complex gene networks, such as the bistable switch [18], the fim switch [19], the oscillator [20] and the *lac *operon [21].

Using computer simulations, we aim to facilitate rational synthesis of tetracycline-inducible regulatory networks and propose new designs that aim to address some of the limitations, for example enable tighter control of expression. We first generate four novel gene regulatory networks based on the tTA, rtTA and the wild type operon of *E. coli*. Then a chemical kinetics model based on the interactions present in the network is constructed and the dynamical behavior of the wild type network is simulated. The model consists of all distinct biomolecular interactions involved in transcription, translation, regulation and induction. The behavior is evaluated and design principles, such as mutations, are introduced, which aim in a fine tuned dynamical behavior. We propose, mutations in TetR sequence which affect both the relative binding affinity with *TetO *[22-24] and with tetracycline [25] allowing for a fine tuned design. Moreover, we suggest mutations in the *TetO *[26-29] sequence that affect the relative binding affinity with TetR.

## Results and discussion

### Four novel networks based on the tetracycline regulated system

**Figure 1:**
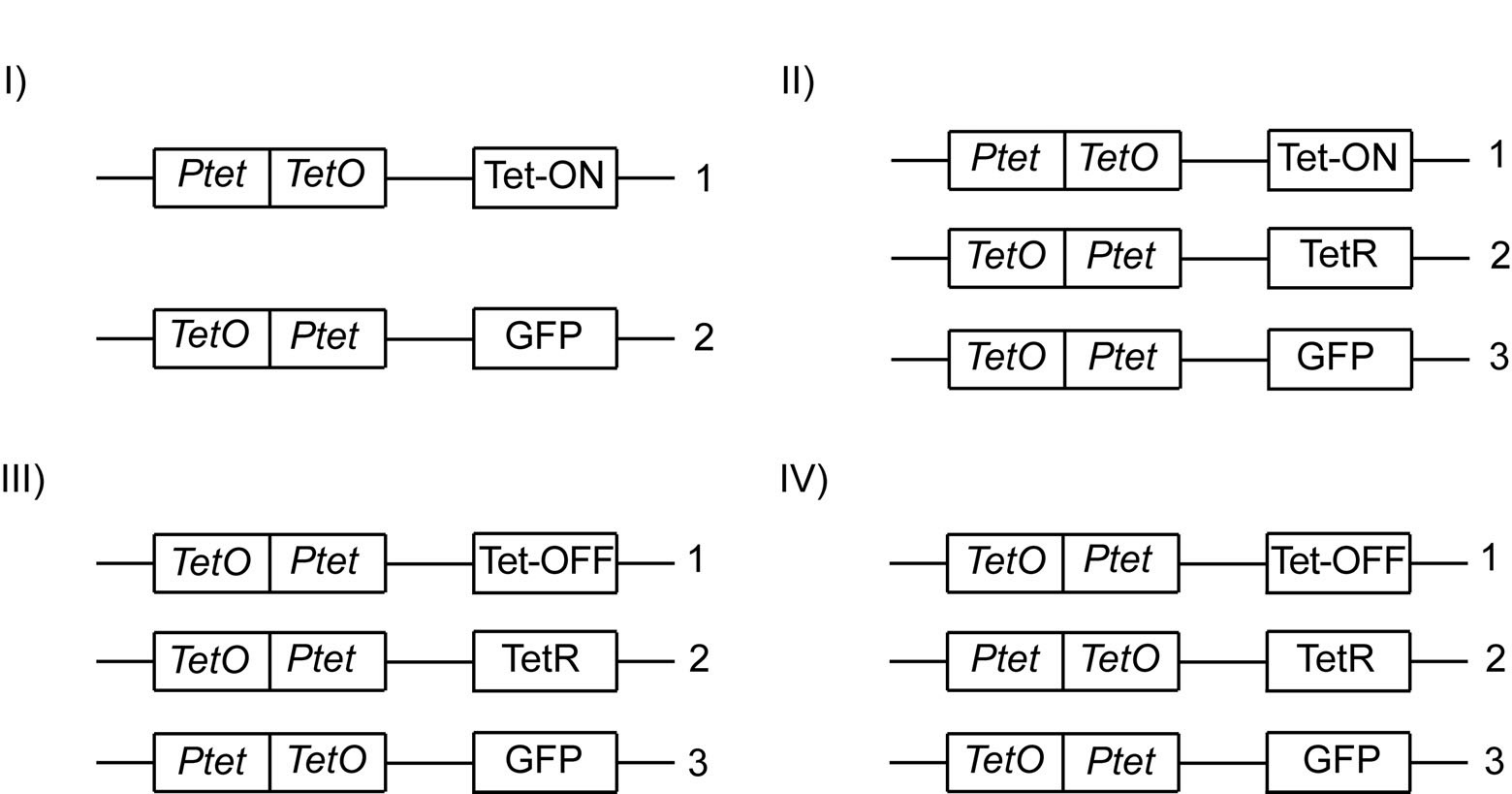
**A schematic representation of four novel regulatory gene networks**. Schematic representation of the four regulatory gene networks showing the way components of the Tet-Off, Tet-On and the tetracycline resistance operon are combined. TetR: the wild type Tet repressor, *TetO*: the wild type *Tet operator*, *Ptet*: the wild type *Tet Promoter*, Tet-OFF: protein fusion of TetR with a transactivator domain, Tet-ON: protein fusion of reverse TetR with a transactivator domain and GFP: Green Fluorescent Protein

**Figure 2:**
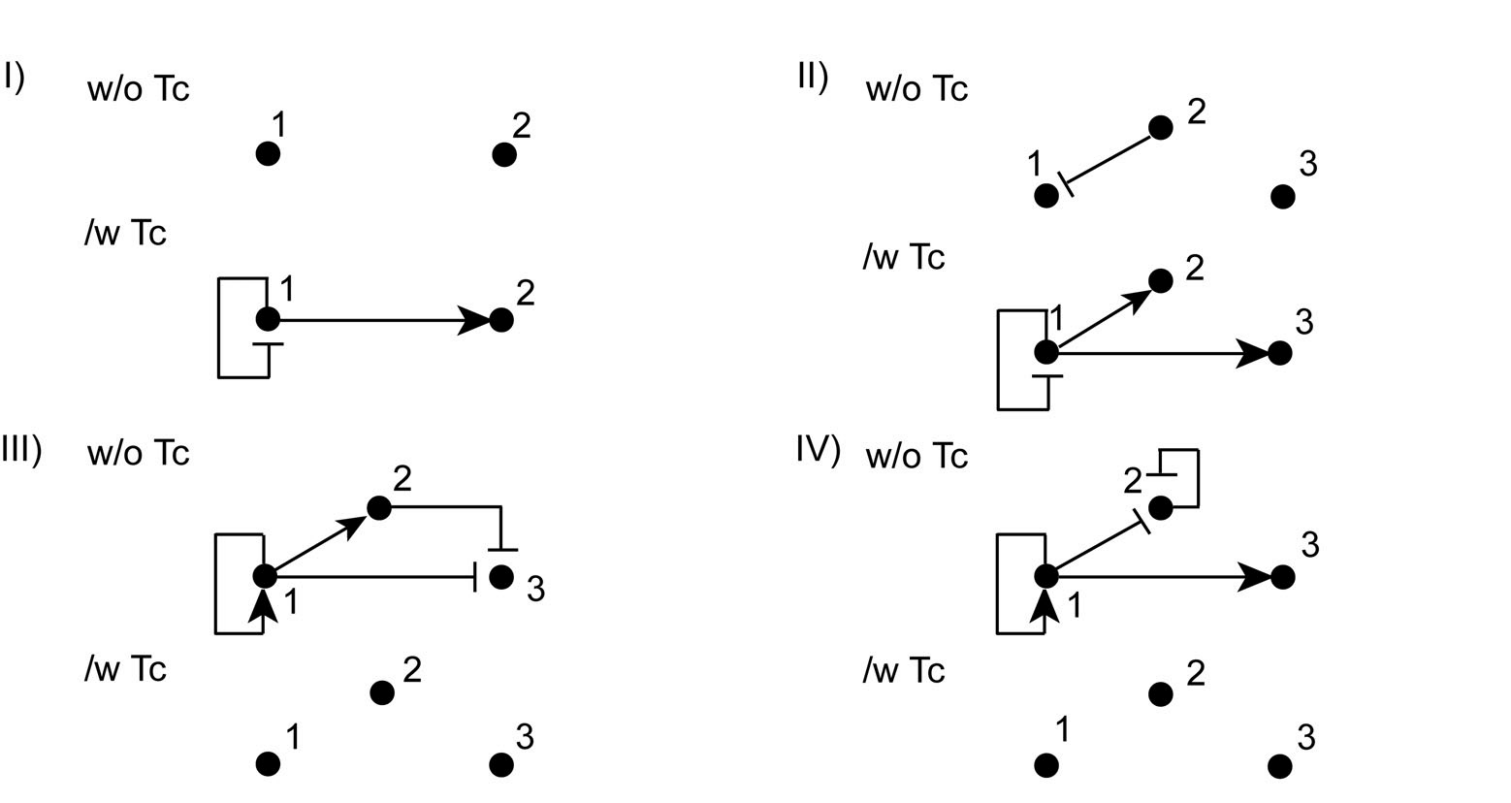
**A schematic representation of the network connectivities**. Schematic representation of the network connectivities in the presence or absence of Tc. Nodes represent genes and are numbered according to Figure 1. Arrows represent repression or activation.

Based on the components of Tet-Off, Tet-On and the tetracycline resistance operon of *E. coli *we introduce four novel model networks that address limitations present in current designs. By computationally identifying the important molecular interactions, the objective is to find ways to fine tune the dynamic response of the systems. We will mainly concentrate in controlling levels of repressor-transactivator proteins, response times and sensitivity to Tc or its analogues. A schematic representation of the proposed gene networks is shown in Figure 1. The connectivity between network components in the absence or presence of Tc are shown in Figure 2, where nodes represent genes and arrows represent repression or activation.

We should point out that the selection of network architectures is a crucial step in model-driven construction of novel gene regulatory networks. In principle, optimization algorithms can be developed that will search for the components (promoters, operators, regulatory proteins, inducers, etc.) and determine optimal configurations of biomolecular interactions, optimizing the dynamic behavior with respect to a set of design objectives. Nonetheless, unless prior knowledge and intuition are used, the problem of computationally designing gene networks ab initio becomes practically intractable.

Based on the building blocks of the wild-type rTA and rtTA we constructed four networks with different number of genes and components, using intuition and knowledge of the dynamic behavior of the components and their interactions. Of course there are a great number of alternative configurations we could have used. But our objective was to keep the proposed networks as simple as possible, keeping in mind what can be experimentally realizable and having a qualitative insight about how close the behavior would be to the desired dynamic phenotype. The problem we concentrated on in this work is the following: given alternative network designs, identify the best solution that meets specific design objectives.

The major components of the four networks are the wild type Tet repressor DNA binding protein (TetR), the wild type *Tet operator *(*TetO*), the wild type *Tet promoter *(*Ptet*), the Tet-OFF protein (fusion of TetR with an appropriate transactivator domain) and the Tet-ON protein (fusion of reverse TetR with an appropriate transactivator domain). Note that the proteins Tet-OFF and Tet-ON are written using capital letters for ON and OFF in contrast to the Tet-Off and Tet-On systems where lower cases are used. When *TetO *is located upstream of *Ptet *we assume that there is no overlapping of the two sequences, with the proximity of the two being appropriate for the transactivators to interact with the transcriptional machinery. The promoter is not silenced while TetR is bound to *TetO*; RNA polymerase can still be recruited. Green Fluorescent Protein (GFP) is used as a reporter gene in our networks. In practical gene therapy applications, a therapeutic gene can replace GFP.

The complexity and the degree of uncertainty we have for mammalian transcription and translation stages prohibit us currently from simulating the gene networks in mammalian cells. Therefore we study the time evolution of the networks within bacteria, in particular *E. coli *where molecular mechanisms are less complex and well studied. We use strings of *E. coli *that do not contain the natural tetracycline resistance operon. Our models incorporate all individual molecular species and interactions known to be involved in the transcription, translation, regulation, and induction steps in the tetracycline-regulated expression system. For example we end up with 93 reactions that model all the individual biomolecular interaction events in Network III. The detailed network of reactions, along with a short description can be found in the Methods section of the present paper. In addition, the model incorporates cell division with the doubling time of cells being 30 ± 4 min. Averages are computed from 100 independent trajectories using the Hybrid Stochastic Algorithm (Hy3S) for each network of reactions (see Methods section).

We start by looking into the wild type dynamical behavior of each network, using chemical kinetics models. This approach allows us to look into the molecular level and investigate how species concentrations vary over time and how they affect the actual phenotype. First we determine important interactions and secondly we propose ways to manipulate sequences and binding affinities to achieve design goals. Although intuition can help us to decide what new designs to construct based on qualitative arguments, it is the insight of the molecular level that guides us to propose changes that will attempt to address limitations and also lead to design rules for fine tuned systems. Experimentally realizable changes include the use of new TetR or reverse TetR [22-24] and *TetO *[26-29] variants as well as TetR variants that do not bind Tc [25]. Effects are studied and the suggested changes are accepted, rejected, or combined.

We should stress here, that our objective is not to examine the gene networks simply from a purely mathematical point of view but from a synthetic biologist's point of view. Therefore, most of the changes introduced in the model parameters have to be experimentally realizable. It is important for us if an experimentalist can read our work and go back in the lab convinced that they are indeed feasible. Hence, the parameters altered are chosen so that they represent realistic scenarios. As for the way we decided to explore the available parameter space, this is done in a discrete manner. We did not use a sensitivity analysis because we attempt to realistically model cells and this immediately implies that not all parameter space is available. This is because there is no way we can change the range of a parameter continuously by introducing proper mutations in the coding and/or DNA sequences. Changes in the sequence of amino acids results in a discrete change in the corresponding kinetic values and hence a sensitivity analysis, though useful for modeling purposes, would likely not add any greater insight in the construction of such networks in a Petri dish.

### Network I

#### Dynamical behavior based on wild type kinetics

Starting with Network I (Figure 1), we intend to control the concentration of Tet-ON with self-repression and decrease the sensitivity of the network to low Tc levels. This decreased sensitivity will result in shorter time intervals before gene expression of the reporter gene is turned back off again, after Tc administration. With Tc present, Tet-ON can bind on *TetO*, downstream of *Ptet *and self-repress while transcription of the reporter gene is on and activated by Tet-ON. The rate of the transcription depends on the amount of Tet-ON induced with Tc available and on the promoter strength. In the absence of Tc, Tet-ON does not bind to either *TetO *sequences and expression levels of Tet-ON and GFP will depend on promoter strength (basal activity). For small Tc concentrations, self-repression of Tet-ON will result in a decrease of Tet-ON concentration and lower expression of reporter gene.

**A schematic representation of these interactions can be seen in Figure **2A

**Figure 3:**
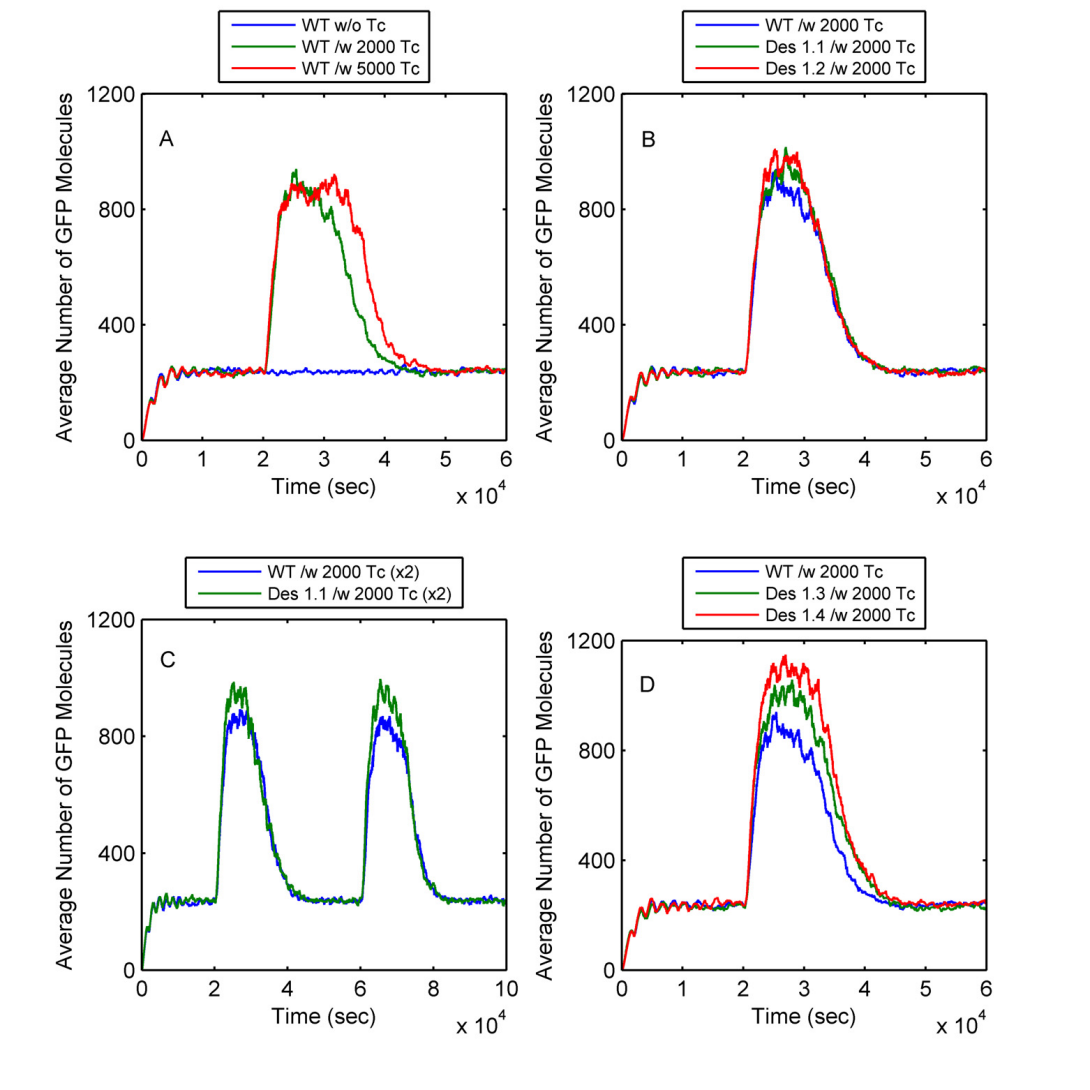
**Dynamical behavior of Network I**. (A) Average number of GFP molecules in the absence of Tc (WT w/o Tc, blue line) and when 2000 molecules (WT/w 2000 Tc, green line) or 5000 molecules (WT/w 2000 Tc, red line) of Tc are added into the medium at time 2 × 10^4 ^sec, using wild-type kinetics. (B) Average number of GFP molecules when 2000 molecules of Tc are added into the medium at time 2 × 10^4 ^sec, using wild-type kinetics (WT/w 2000 Tc, blue line), a 20 fold (Des 1.1/w 2000 Tc, green line) and a 50 fold (Des 1.2/w 2000 Tc, red line) increase in the dissociation constant of induced Tet-ON from *TetO *of the gene encoding Tet-ON. (C) Average number of GFP molecules when 2000 molecules of Tc are added into the medium at time 2 × 10^4 ^sec and 6 × 10^4 ^sec, using wild-type kinetics (WT/w 2000 Tc (x2), blue line), a 20 fold (Des 1.1/w 2000 Tc (x2), green line) increase in the dissociation constant of induced Tet-ON from *TetO *of the gene encoding Tet-ON. (D) Average number of GFP molecules when 2000 molecules of Tc are added into the medium at time 2 × 10^4 ^sec, using wild-type kinetics (WT/w 2000 Tc, blue line), a doubled (Des 1.3/w 2000 Tc, green line) and a quadrupled (Des 1.4/w 2000 Tc, red line) half-life of Tet-ON.

First we investigate the dynamical behavior, both transient and at equilibrium, of the network. The dynamical behavior of Network I in the presence or absence of Tc, over a time period of 6 × 10^4 ^sec (16.7 hours) is presented in Figure 3A. In the absence of Tc basal expression of GFP is approximately 250 molecules. The system reaches an equilibrium state after10^4 ^sec (2.8 hours) with Tet-ON dimer concentrations values of approximately 40 molecules (data not shown). On the other hand, GFP production is increased when Tc is added, 2000 and 5000 molecules at time 2 × 10^4 ^sec (Figure 3A). Maximum GFP values reach levels of approximately 850 molecules, a 200 % increase from basal expression. The differences between the two cases are the time that the system sustains maximum levels of GFP and eventually the turning off time. This differentiation is a direct result of Tet-ON dimer concentration before the addition of Tc and the concentration of added Tc. In both cases, before adding Tc the concentration of Tet-ON monomers and dimers was the same and that led to similar levels of induced Tet-ON, meaning same GFP values. The lengthened duration of the pulse in case two is mainly due to the larger amount of free and available Tc molecules that sustained induced Tet-ON molecules longer. In both cases maximum free Tc amounts in the cell were below toxicity levels, approximately 600 (0.44 μg/mL) and 1600 (1.18 μg/mL) molecules respectively.

#### Fine tuning using mutated TetR and TetO variants

As we observe the system experiences high levels of basal expression. This has been anticipated since the *Ptet *promoter is naturally a strong promoter. Overcoming this limitation will require to change *Ptet *with a minimal one, something attempted successfully in mammalian cells with a promoter from hCMV. For this we will focus on other strategies to fine tune the system, such as mutating operator sequences and changing half-lifes.

The first proposed change is to mutate *TetO *of the gene encoding Tet-ON. Mutating *TetO *in general will result in less binding affinity for induced Tet-ON, since TetR or revTetR interaction with *TetO *is considered to be very strong. Therefore we will consider only cases where a decrease in the binding affinity is observed. Implementing the change in our model requires increasing the dissociation constant of the induced Tet-ON dimer from *TetO *(decrease half-life of the complex). Initially, the wild type kinetic constant was set to 0.01 sec^-1^. We changed the dissociation kinetic constant from 0.01 sec^-1 ^to 0.2 sec^-1 ^and then again to 0.5 sec^-1^. The results are shown in Figure 3B, where at time 2 × 10^4 ^sec there is an addition of 2000 molecules of Tc in the medium. Although we observe a decrease in the occupancy of the mutated operator and a small increase in the number of induced Tet-ON dimers as well as the time period they are present, no significant change in the occupancy of the operator in the reporter gene is observed. Therefore as the kinetic parameter is increased, GFP levels are slightly altered whereas pulse duration practically remains the same. This becomes more evident in Figure 3C where we compare the dynamical behavior of the wild type kinetics with the operator having a 20 fold (0.2 sec^-1^) decrease in the affinity for Tet-ON.

On the other hand mutating the operator adjacent to *Ptet *encoding GFP will only result in decreased production of GFP, a direct consequence of the reduced occupancy of the operator. The same outcome can be achieved by adding less Tc into the system.

Another way to fine tune the system's behavior is by increasing the half-life of Tet-ON, leading to higher amounts of Tet-ON before administration of Tc. Applying such a change requires the addition of a C-terminal tag. The half-life of Tet-ON was initially set to 10 min; we looked into the cases of doubling it (5.7762 × 10^-4 ^sec^-1^) and quadrupling it (2.8881 × 10^-4 ^sec^-1^). The results are shown in Figure 3D, where again 2000 molecules of Tc are added at 2 × 10^4 ^sec. In both cases we observe a change in the phenotype, GFP levels are increased over time and the duration is also increased. Looking into the levels of free and induced Tet-ON molecules we observe an increase of almost 75 % and a 150% for doubled and quadrupled half-lifes, respectively. Comparable increases in GFP levels or duration are not monitored, due to non significant alternation in the occupancy of *TetO *in the reporter gene.

Concluding, controlling GFP levels and duration of the pulse (turning on and off times) cannot be accomplished separately. Both are related to the amount of Tet-ON dimers in the system prior to inducer's administration and on the amount of the inducer added. The higher the amounts of Tet-ON dimers and of inducer, the higher GFP levels are going to be. On the other hand longer duration is achieved, by keeping the levels of induced Tet-ON constant over time.

### Network II

#### Dynamical behavior based on wild type kinetics

In Network II (Figure 1), we add a third gene encoding TetR to improve the regulation achieved with the first design. In the absence of Tc, TetR will minimize expression of all genes including itself. In the presence of Tc, TetR will no longer repress. Tet-ON levels will increase depending on the strength of self-repression, activating GFP expression. Tet-ON will also activate expression of TetR. At low levels of Tc, TetR binds to Tc and represses Tet-ON and reporter gene expression.

**Schematically these interactions are shown in Figure **2B

**Figure 4:**
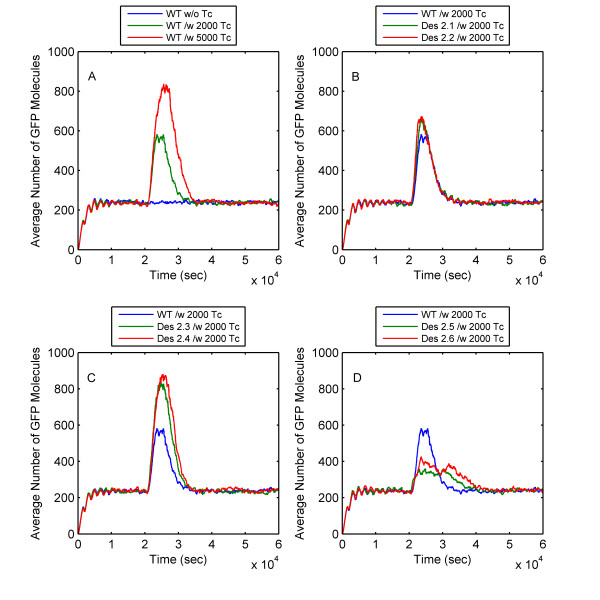
**Dynamical behavior of Network II**. (A) Average number of GFP molecules in the absence of Tc (WT w/o Tc, blue line) and when 2000 molecules (WT/w 2000 Tc, green line) or 5000 molecules (WT/w 5000 Tc, red line) of Tc are added into the medium at time 2 × 10^4 ^sec, using wild-type kinetics. (B) Average number of GFP molecules when 2000 molecules of Tc are added into the medium at time 2 × 10^4 ^sec, using wild-type kinetics (WT/w 2000 Tc, blue line), a 10 fold (Des 2.1/w 2000 Tc, green line) and a 50 fold (Des 2.2/w 2000 Tc, red line) increase in the dissociation constant of both TetR and induced Tet-ON from *TetO *of the gene encoding Tet-ON. (C) Average number of GFP molecules when 2000 molecules of Tc are added into the medium at time 2 × 10^4 ^sec, using wild-type kinetics (WT/w 2000 Tc, blue line). All other plots have a 10 fold increase in the dissociation constant of both TetR and induced Tet-ON from *TetO *of the gene encoding Tet-ON, but differ in a 5 fold (Des 2.3/w 2000 Tc, green line) and 20 fold (Des 2.4/w 2000 Tc, red line) increase in the dissociation constant of both TetR and induced Tet-ON from *TetO *of the gene encoding TetR. (D) Average number of GFP molecules when 2000 molecules of Tc are added into the medium at time 2 × 10^4 ^sec, using wild-type kinetics (WT/w 2000 Tc, blue line). All other plots have mutated TetR variants that do not bind Tc and show a decreased binding affinity for *TetO *(20 fold decrease in the dissociation constant), but differ in the half-life of Tet-ON, 40 min (Des 2.5/w 2000 Tc, green line) and 24 hr (Des 2.6/w 2000 Tc, red line).

Adding a third gene in the first network encoding TetR actually makes the system less sensitive to Tc concentrations. Figure 4A compares the behavior of the system when there is no inducer present and when we add Tc, 2000 and 5000 molecules respectively at time 2 × 10^4 ^sec. If we further compare these results with the ones presented in Figure 4A we observe that the system exhibits the same phenotype when Tc is absent, while GFP levels are down and the duration of the pulse is smaller when the inducer is present. Network II has an extra source of Tc consumption, TetR dimers, so there is less free and available Tc concentration to induce Tet-ON. Also there is one extra *TetO *competing for the transactivator as well as Tet-ON has to compete with TetR for *TetO*. Concentrations of induced TetR reach maximum levels of approximately 140 and 180 molecules, for addition of 2000 and 5000 molecules of Tc respectively. On the contrary, induced Tet-ON molecules reach maximum levels of about 28 and 35 molecules respectively, lower compared to Network I. These lower values, together with the presence of TetR account for less GFP production. Comparing the different scenarios of inducer administered we notice that the more Tc present the more GFP is produced and for a longer period. At last, maximum free Tc amounts in the cell were below toxicity levels, approximately 400 (0.30 μg/mL) and 1200 (0.89 μg/mL) molecules, respectively.

#### Fine tuning using mutated TetR and TetO variants

It can be again observed that the system experiences high levels of basal expression. As mentioned before, a plausible solution for *E. coli *is the use of a minimal promoter, while in mammalian cells rTS could substitute TetR. In the remaining section the focus is to improve the design by proposing strategies, for instance mutated operator sequences or coding sequences that alter the dynamical behavior.

First we start by introducing a mutation in the operator controlling the expression of Tet-ON. This change will affect both TetR and induced Tet-ON binding. For simplicity we assume that the change is analogues for both cases. The approach is indeed simplified, but this assumption is made due to the similarity of the two proteins that differ only by a small number of mutations. The idea behind this mutation is to decrease the turning on response time but also increase GFP levels. TetR dimers will bind weaker, resulting in higher Tet-ON dimer levels at equilibrium. At the same time self repression is limited in the presence of the inducer. We changed the dissociation kinetic constants from 0.01 sec^-1 ^(wild-type) to 0.1 sec^-1 ^and then again to 0.5 sec^-1 ^in the case of induced Tet-ON and from 5.11 × 10^-4 ^sec^-1 ^(wild-type) to 5.11 × 10^-3 ^sec^-1 ^to 2.555 × 10^-2 ^sec^-1 ^in the case of TetR. The results are depicted in Figure 4B, where at time 2 × 10^4 ^sec there is an addition of 2000 molecules of Tc. As the affinity decreases, we observe higher levels of GFP but also a small but visible decrease in the turning on time. Increased Tet-ON levels at equilibrium help the system to respond faster when Tc is added. Indeed, levels of Tet-ON dimer prior to Tc administration show a 500 % and 1250% increase for a 10 fold and 50 fold increases in the dissociation constants, respectively. In contrast, the actual phenotype is only increased by 15% (from approximately 570 to 660 molecules of GFP), since the actual increase of induced Tet-ON dimers is only 25% for both cases.

We also propose to mutate *TetO *in the gene encoding TetR. The objective is to decrease production of TetR when Tc is added. The expectations are to observe higher expression of GFP and less Tc bound TetR. The latter is a consequence of the fact that less TetR is being produced when the inducer is present, while the former is a result of higher transactivator concentrations. Results are not shown for brevity but the objectives are largely met. Furthermore, the system appears to have a small increase in the turning off time.

Since both above mentioned mutations were in the same direction, the next logical step is to combine them. In Figure 4C, the wild type kinetics dynamical behavior is compared to the behavior of the mutated *TetO*'s. In all cases, the dissociation constants concerning *TetO *of the Tet-ON gene where increased by a factor of 10, while those for the other *TetO *have a 5 or a 20 fold increase. From the figure it is obvious that levels of GFP are up, the turning off time is also increased while the turning on time is shortened. Apparently, the two mutations acted additively in the case of GFP production. The mutated *TetO *of TetR increased the turning off time whereas the other mutated *TetO *contributed to the decreased turning on time. Obviously, one can adjust the parameters accordingly in order to achieve the tergeted phenotype.

Finally, a more radical approach is attempted. The wild type behavior is compared with the ones resulting from a series of mutations (Figure 4D). First TetR is mutated so that it does not bind Tc and at the same time mutated to bind weaker to all *TetO*, a 20 fold increase in the dissociation constant. The two mutations do not overlap in the coding sequence, since different amino acids are responsible for the DNA binding and for Tc binding. For simplicity we assume there is no direct or indirect (allostery) interference between the two mutations. Second, the half-life of Tet-ON is increased from 10 min to 40 min and then again to 24 hr. Briefly the idea is first to increase Tet-ON concentration before the addition of Tc and to reduce the need for Tc. The results are inferior if the interest is in GFP production but on the other hand the duration of the pulse is increased.

Concluding we observed that by adding TetR in the equation we are able to adjust and better control the expression of GFP. We are able to regulate both turning on and off times and at the same time manipulate levels of GFP. The downside is that for a given addition of Tc concentration Network I will reach higher GFP levels compared to Network II, since the latter has an extra source of Tc consumption, namely TetR.

### Network III

#### Dynamical behavior based on wild type kinetics

With Network III we anticipate to increase sensitivity to Tc. Without Tc, Tet-OFF production is on and self-activating. Tet-OFF also activates TetR expression. Furthermore, TetR production is also on but self-repressing and at the same time TetR represses Tet-OFF and GFP expression. In this network, Tet-OFF represses expression of the reporter gene instead of activating it. TetR stimulates the amount of both TetR and Tet-OFF dimers in the cell by competing with Tet-OFF for *TetO*. In the presence of Tc, GFP levels will mainly depend on the basal expression of the promoter and the ratio of Tc over Tet-OFF and TetR concentrations.

**Figure **2C** summarizes the interactions betweens genes in Network III**

**Figure 5:**
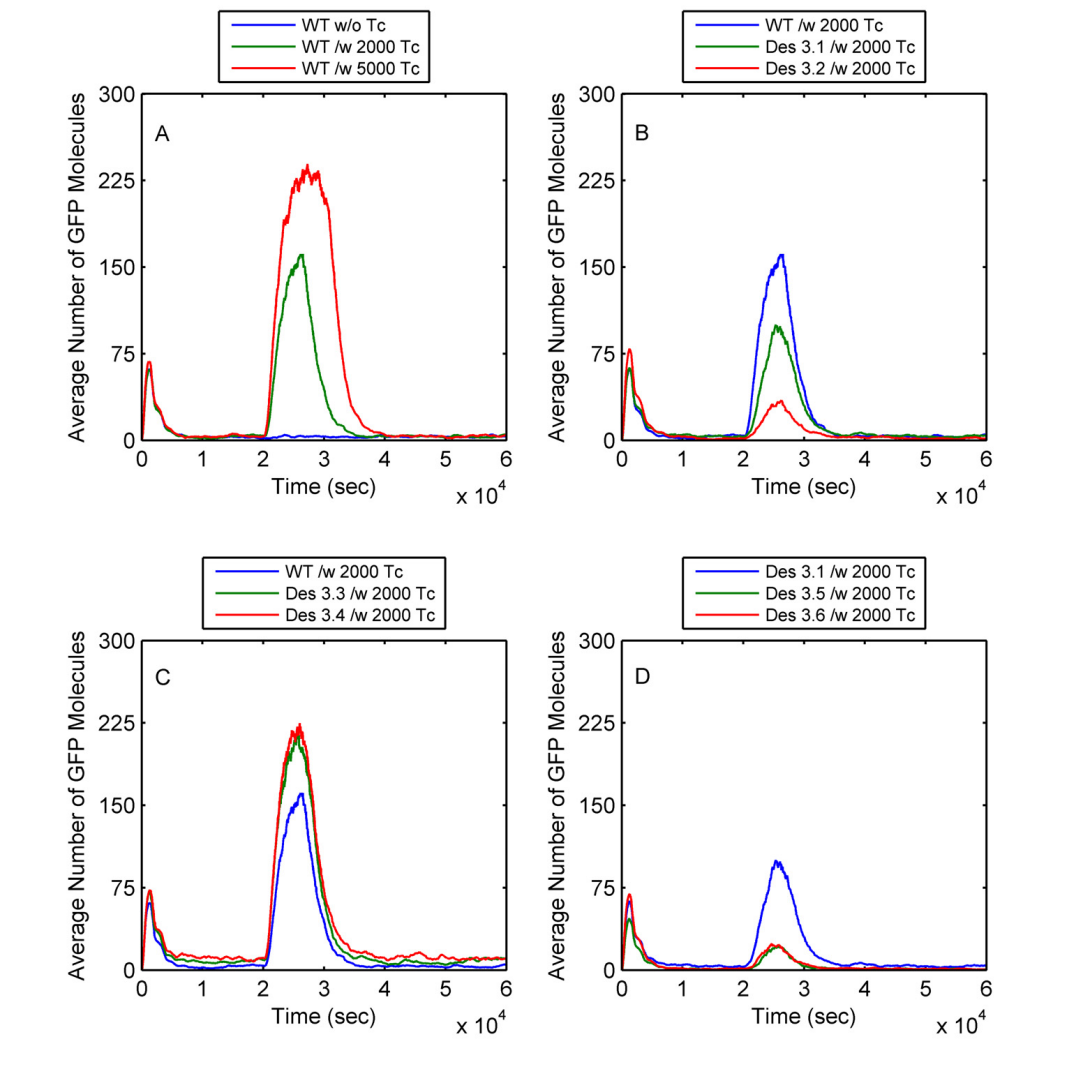
**Dynamical behavior of Network III**. (A) Average number of GFP molecules in the absence of Tc (WT w/o Tc, blue line) and when 2000 molecules (WT/w 2000 Tc, green line) or 5000 molecules (WT/w 5000 Tc, red line) of Tc are added into the medium at time 2 × 10^4 ^sec, using wild-type kinetics. (B) Average number of GFP molecules when 2000 molecules of Tc are added into the medium at time 2 × 10^4 ^sec, using wild-type kinetics (WT/w 2000 Tc, blue line), a 10 fold (Des 3.1/w 2000 Tc, green line) and a 50 fold (Des 3.2/w 2000 Tc, red line) increase in the dissociation constant of TetR for all *TetO *(mutated TetR variant). (C) Average number of GFP molecules when 2000 molecules of Tc are added into the medium at time 2 × 10^4 ^sec, using wild-type kinetics (WT/w 2000 Tc, blue line), a 5 fold (Des 3.3/w 2000 Tc, green line) and a 20 fold (Des 3.4/w 2000 Tc, red line) increase in the dissociation constant of Tet-OFF for all *TetO *(mutated Tet-OFF variant). (D) Average number of GFP molecules when 2000 molecules of Tc are added into the medium at time 2 × 10^4 ^sec, using for all simulations a mutated TetR variant with 10 fold (Des 3.1/w 2000 Tc, blue line) increase in the dissociation constant plus a tripled (Des 3.5/w 2000 Tc, green line) and a quadrupled (Des 3.6/w 2000 Tc, red line) half-life for both Tet-ON and TetR.

Simulating the time evolution of Network III (Figure 5A) using wild-type kinetics, results in a substantially different observed phenotype. In the absence of Tc, equilibrium state values of GPF approach zero, approximately 4 molecules of GFP. A sharp pick in the concentration of GFP in the transient period is a result of small initial TetR and Tet-OFF concentrations, which at equilibrium sum up to a total average number of approximately 300 molecules. Next we add 2000 and 5000 molecules of Tc at 2 × 10^4 ^sec (Figure 5A). The higher the inducer concentration, the more time the operator, located downstream in the reporter gene, will be unoccupied leading to more GFP for longer time periods. GPF maximum concentrations values approach levels of approximately 250 molecules (basal expression). This network takes into account and effectively uses the high basal expression of *Ptet*. Again Tc levels remained below toxicity levels.

#### Fine tuning using mutated TetR and TetO variants

The challenges that this network poses are first to eliminate expression leaking when Tc is absent and second, to increase the sensitivity of the network to Tc. Beginning with the first challenge an obvious step is to increase "repressors" levels, meaning the total amount of both TetR and Tet-OFF dimmer molecules, when Tc is absent. This can be accomplished if we allow Tet-OFF to occupy operator sites for longer times compared to TetR. One alternative is to mutate TetR so that it shows weaker binding to *TetO*. Increasing the dissociation of TetR from *TetO *by a factor of 10 or 50, we managed indeed to achieve a decrease in the levels of GFP, but we did not manage to make them zero (Figure 5B). Levels of "repressors" indeed raised 50 % and 100 % for a 10 and 50 fold increase in the kinetic parameter, respectively. To generalize, as TetR binds progressively more weakly to *TetO*, GFP levels in the absence of Tc decrease by two molecules (50%) when the dissociation constant is increased 50 times. Conversely, GFP levels decrease drastically for a given (2000 molecules of Tc added at 2 × 10^4 ^sec) concentration of inducer, less sensitivity to Tc.

On the other hand, trying to increase the sensitivity of the system to Tc concentrations requires the opposite, a decrease in the "repressors" concentration. Similarly, mutating Tet-OFF instead of TetR, leads to smaller production of both proteins. In Figure 5C we compare the wild type phenotype with the one observed by increasing the dissociation constant 5 (2.555 × 10^-3 ^sec^-1^) and 20 (1.022 × 10^-2 ^sec^-1^) times. It is obvious that GFP levels increase towards basal expression levels. "Repressors" levels in the cell decrease approaching total values of 80 molecules, with leaking becoming more evident in the absence of inducer.

Since the above two mutations do not point on the same direction, it would not be fruitful to try to combine them. For this we tried something more extreme in order to eliminate leaking. We mutated TetR for smaller binding affinity to *TetO *sequences and also we increased the half-life of both TetR and Tet-OFF with the purpose of increasing the overall concentration of "repressors". We keep the same TetR mutant in all simulations presented, 10 fold increase in the kinetic parameter, while we triple, (30 min) and quadruple (40 min) the half-lifes. The results are shown in Figure 5D. The increase in the "repressors" total concentration is approximately 100 molecules (22%) for both half-life cases whereas the actual decrease in GFP is 3 molecules (75 %). In Conclusion, increasing the half-life eventually will lead to zero GFP levels but with large amounts of proteins molecules in the cell that may be toxic. At the same time large inducer amounts are required to transfer the system from the "Off" to the "On" state.

In conclusion we explored possible mutations that would allow us to eliminate expression leakage. We observed that even if we increased "repressor" molecules levels by 100% leaking is still present but in limited amounts. For complete silencing large amounts of "repressor" molecules are required leading to toxicity concerns. On the other hand increasing sensitivity to Tc requires less "repressor" molecules being present. Therefore depending on application requirements we can adjust the system parameters in order to achieve either very low GFP expression or higher sensitivity.

### Network IV

#### Dynamical behavior based on wild type kinetics

Finally for Network IV in the absence of Tc, Tet-OFF production is on and self-activating and TetR production is also on, but self-repressing. GFP expression will also be on, but how strongly depends on the ratio of TetR and Tet-OFF amounts available. With Tc present, TetR production is on, Tet-OFF and reporter gene production depend on the promoter strength. A schematic representation of these interactions can be seen in Figure 2D. Note that constant Tc administration will be required for the expression to be silenced, a limitation following Tet-OFF. Adjusting turning on response times is the objective in the present network.

**Figure 6:**
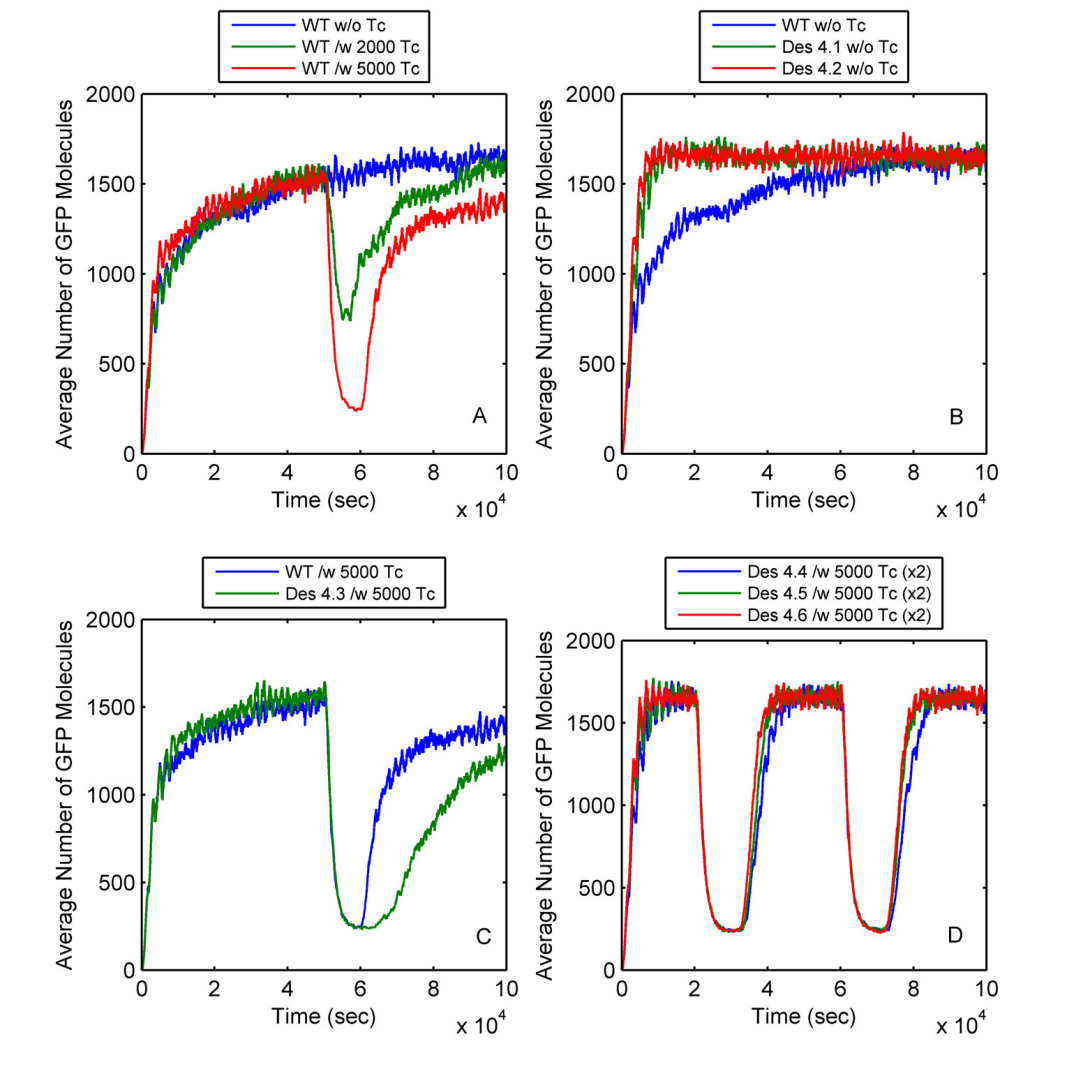
**Dynamical behavior of Network IV**. (A) Average number of GFP molecules in the absence of Tc (WT w/o Tc, blue line) and when 2000 molecules (WT/w 2000 Tc, green line) or 5000 molecules (WT/w 5000 Tc, red line) of Tc are added into the medium at time 5 × 10^4 ^sec, using wild-type kinetics. (B) Average number of GFP molecules in the absence of Tc, using wild-type kinetics (WT w/o Tc, blue line), a 10 fold (Des 4.1 w/o Tc, green line) and a 20 fold (Des 4.2 w/o Tc, red line) increase in the dissociation constant of TetR from all *TetO *in the network(mutated TetR variant, less affinity for *TetO*). (C) Average number of GFP molecules when 5000 molecules of Tc are added into the medium at time 5 × 10^4 ^sec, using wild-type kinetics (WT/w 5000 Tc, blue line) and mutated TetR variant that does not bind Tc (Des 4.3/w 2000 Tc, green line). (D) Average number of GFP molecules when 5000 molecules of Tc are added into the medium at times 2 × 10^4 ^sec and 6 × 10^4 ^sec, using a mutated TetR variant that does not bind Tc and also shows different levels of binding affinity for *TetO*, 10 fold (Des 4.4/w 2000 Tc (x2), blue line), 20 fold (Des 4.5/w 2000 Tc (x2), green line) and 50 fold (Des 4.6/w 2000 Tc (x2), red line) decrease in the dissociation constant.

In Figure 6A the time evolution of Network IV is shown. When Tc is absent the network produces higher concentration of GFP than the other networks, equilibrium values are approximately 1650 molecules. Obviously the system has not reached an equilibrium state, even after 28 hours. Addition of Tc causes an evident decrease in GFP production, with the transition from the "Off" to the "On" state having a large response time. This phenotype is a direct consequence of the competition between TetR and Tet-OFF dimers to occupy *TetO *sequences. Initially, or after Tc administration, concentrations of dimer TetR increase rapidly reaching a maximum concentration, only to fall rapidly short thereafter, approaching zero levels. Tet-OFF dimer concentration goes rapidly to 200 molecules and then requires 10 times more time to reach equilibrium values (approximately 300 molecules). These high concentration values are eventually responsible for the increased expression of GFP. High levels of Tc are required in order to drop production of GFP down to basal expression levels. Free maximum Tc concentrations reach levels below toxicity, approximately 100 (0.07 μg/mL) and 1200 0.89 μg/mL molecules for addition of 2000 and 5000 molecules of Tc, respectively.

#### Fine tuning using mutated TetR and TetO variants

By investigating the time evolution of the system, we can pinpoint limitations in the design and propose changes. First, the high basal expression is a common drawback among the proposed networks. Secondly, it is apparent that the response of the system is slow, both initially and after administration of the inducer. Finally, one would like to make the system more sensitive to Tc concentrations for two reasons; easier transition between the "On" and "Off" states and better control over the duration of the "Off" state.

In the previous section, we noted that the slow response is due to competitive binding between Tet-OFF and TetR with *TetO*. Improving the response time will require altering the relative binding affinity of the two dimers. We mutate TetR, since it is the one that does not add to GFP production. The appropriate kinetic parameters were altered from 5.11 × 10^-4 ^sec^-1 ^to 5.11 × 10^-3 ^sec^-1 ^to 1.022 × 10^-2 ^sec^-1^, a 10 and 20 fold increases respectively. Simulating the new network we find that the initial lag is reduced significantly (Figure 6B). The system reaches equilibrium values in only 2.8 hrs. Note that the difference in the responses between the two mutations is small, which means that a little alternation is capable of producing the targeted behavior. Additionally, Tet-OFF levels reach their equilibrium values much faster than before. The difference is also noted by looking at the percent of Tet-OFF occupying *TetO*'s over time, increasing as TetR affinity for *TetO*'s decreases.

In this particular system, increased sensitivity to Tc can be achieved through a decrease in the equilibrium values of TetR and Tet-OFF. However, this will also cause a decrease in GFP levels. Another way to go about this problem is to use TetR variants that do not bind to Tc. The downside is that TetR will always be able to bind to *TetO *sequences. Using the last alternative, we simulate the system and the results are presented in Figure 6C. Obviously the new system appears to have longer pulse duration. Still the response time for transition between "On" and "Off" states remains large.

Since both mutations in the coding sequence of TetR improved the design we decided to combine them, assuming the two mutations do not interfere with each other. In Figure 6D the time evolution of the system is presented. Tc is added into the system at two time points, 2 × 10^4 ^sec and 6 × 10^4 ^sec. Though all TetR variants have no affinity for Tc, they have different levels of binding affinity for *TetO*, namely 10, 20 and 50 fold increase in the dissociation constant. Indeed the behavior of the system looks superior compared to the wild type. Furthermore, the network exhibit shorter turn off times as the binding affinity of TetR for *Tet*O weakens. Constant Tc administration for low GFP production as well as the high GFP production in the absence of inducer (560% above basal expression) render this system difficult but at the same time attractive for applications.

In summary, we achieved to decrease the response times of the network in both the transient period and also after Tc administration. Adjusting the corresponding kinetic parameter gives the required edge to Tet-OFF over TetR and hence improves the response. Additionally we explored ways to decrease the necessity for Tc in order to "silence" the system. We observed that by mutating TetR appropriately the required amounts of Tc are indeed reduced.

## Conclusion

Using all the molecular components of transcription, translation, regulation and induction, the dynamic behavior of the proposed synthetic gene networks can be simulated and screened for possible improvements. It should not go unnoticed that the simulation of a system that spans many orders of magnitude in kinetic constant values is indeed realizable. To achieve this, we use a hybrid dynamic stochastic-discrete and stochastic-continuous algorithm equipped with an adaptive time stepping method for numerically integrating the set of stochastic differential equations in the model. The simulations allow the quick and inexpensive investigation and comparison of multiple alternative designs. They provide a clear insight at the molecular level, while experiments focus on the phenotype. The key is to identify the important interactions and based on them propose design rules. Important interactions can be both obvious but non-apparent in terms of their impact on the phenotype of the system. Ideally, the computational approach will be able to investigate thoroughly all possible alternatives and adjust the dynamical behavior of a gene network to fit certain demands.

Based on the tetracycline-regulated systems, we propose four novel regulatory gene networks in order to alleviate limitations faced in widely used systems. We improved the design of all networks using mutations in the coding and operator sequences. Though there is still plenty of room for improvement, especially if one considers the amount of available operator, promoter and coding sequences that exist in nature. The near perfect adaptation quality observed is inherent to the composition of these network: tetracycline binds very strongly and fast on the regulatory proteins and, in turn, Tet-Off and Tet-On unbind and bind opearator sites respectively, again with remarkable robustness in terms of strength and speed. This behavior is observed in experiments of Tet-On and Tet-Off and is also observed in the simulated results. The dynamic phenotype of these systems is what makes them appealing building blocks for robust, useful switches. Our model-driven designs can become the first step in improved gene regulatory networks.

## Methods

### Chemical kinetics models

Representing the physicochemical interactions between biomolecules, such as recruitment of RNA polymerase on promoter sites, as a set of chemical reactions enables us to study the time evolution of a gene network using stochastic algorithms. The knowledge for the molecular mechanism of transcription and translation provides us with enough insight to classify interactions in the molecular level as first order, second order, Michaelis Menten type, etc. reactions. Reversible phenomena, as binding and unbinding of tetracycline to TetR, are represented as two separate reactions (forward and reverse reactions). Special events as transcriptional elongation are modeled as gamma distributed events [30], whereas interactions between three or more species, where one of the species has a binary state, (one or zero number of molecules, for instance non occupied and occupied operators) are assumed to follow power law kinetics.

As an example, consider the synthetic Network III (see sections above). The network consists of 63 species, participating in 93 distinct chemical reactions. In Table 1, we present all reactions with their kinetic parameters. These parameters are largely taken from the existing literature and others adjusted to give specific values, for example the rates of mRNA half-life and mRNA ribosome binding (initialization of translation) are adjusted to produce approximately 20 protein molecules per mRNA transcript. Table 1 represents the wild-type behavior of the genes. We will briefly describe how we assigned the appropriate kinetic data to the set of reactions. For brevity we will focus on the reactions depicted by design III (Table 1), but the approach is similar for the rest of the networks.

Administration of Tc into the medium causes diffusion through the cytoplasmic membrane of *E. coli *[31]. The process has a half-equilibration time of approximately 35 ± 15 min and is modeled as first order chemical reaction (k = 3.3 × 10^-4 ^sec^-1^).

Dimerization of TetR and Tet-OFF are reversible reactions and their equilibrium constants, in the absence of specific experimental information, are assumed to be similar to *lac *[32]. Binding of tetracycline to TetR is also a reversible phenomenon and equilibrium constants are readily available in the literature [4,33]. In the case of Tet-OFF, we assume it has the same binding affinity for Tc as TetR, a reasonable assumption if one notes that the inducer binding domain of TetR is not affected when the transactivator is added. Each TetR or Tet-OFF dimer requires two molecules of Tc to be fully induced. Due to the stochastic nature of the algorithm it is in general difficult to model a reaction where more than two species are simultaneously involved. We break down the reaction of the two Tc molecules with either one TetR or Tet-OFF dimer into two steps. In the first step, one Tc molecule reacts with one TetR/Tet-OFF dimer molecule with rate constant 2.0 × 10^6 ^(M sec)^-1 ^and in the next step the formed complex reacts very fast (1.0 × 10^15 ^(M sec)^-1^) with another Tc molecule to form the fully induced complex. It is obvious that the first step is the rate limiting one and that the underlying assumption is that Tc induction depends linearly on the concentration of Tc. Finally, due to the short life of the intermediates we do not consider them degrading.

Binding constants for TetR and Tet-OFF dimers in the operator sequence (*TetO*) are available in the literature [33,34]. As previously, we assume a similar behavior between the two dimers. Presence of Tc causes TetR or Tet-OFF dimmers bound to an operator to unbind faster [35], meaning that that the complexes have a smaller half-life (2 min) as compared to the normal half-life of approximately 20 min. Similar to the binding of two Tc molecules to free TetR, the reaction of two Tc molecules with bound to an operator TetR is again broken down to two steps.

Protein degradation can be modelled as a first order reaction, with the kinetic constant calculated from half-life data. Protein half-lifes can vary by many orders of magnitudes and depend on the cell type and environmental conditions. Consequently it would be invalid to consider a typical value that would apply universally. The solution to this problem comes by adding a C-terminal tag. In the present study we assumed that all proteins, except GFP, have an initial half-life of approximately 10 min (0.0012 sec^-1^). Wild type GFP degradation is slow, has a half-life of approximately 26 hours. For distinct turn on and off times of the reporter gene smaller half-life times are desired. New unstable variants of GFP proteins have been introduced [36]. We choose GFP-L-A-A (the last three letters denote the amino acids of the C-terminal tag), which has a reported half-life of 40 min. Finally, since for *E. coli *there is no specific pathway for biodegrading Tc, we assume that the rate at which Tc is removed from the system is equal to the half-life (48 hrs) of Tc in distilled water [37].

*E. coli *RNA polymerase recruitment to the promoter region, interaction with the occupied or not operator region, formation of close complex and then formation of the open complex are modelled through a cascade of reactions. Literature data [38,39] provide the desirable kinetic constants. Open complex formation is assumed to be irreversible, since cells try to minimize their energy use. Transactivators in general attract, position and modify RNA polymerase. Given that *Ptet *is a strong promoter we assume that the presence of either Tet-OFF or Tet-ON in close proximity to the promoter mainly affects the formation of the open complex. In the present study we assume that the kinetic constant of the irreversible formation of the open complex is increased by a factor of 10, since we were not able to obtain kinetic data for prokaryotic transactivators.

Recruitment of RNA polymerase is described using power law kinetics. Initiation of transcription is modeled as a first order reaction, whereas elongation is considered to be a gamma distributed event [30]. Movement of the RNA polymerase across the DNA (coding sequence), occurs at a rate of approximately 30 nucleotides/sec [40]. The parameter N of the gamma distribution is equal to the number of nucleotides each coding sequence has. TetR is comprised of 207 Amino Acids (AA), whereas Tet-OFF has the extra AA from the transactivator domain. The GFP variant is comprised of 238 AA, plus three AA from the peptide chain.

As in the case of proteins, mRNA can be degraded. Similar to proteins there are complex pathways for biodegrading mRNA. Again we considered degradation to be a first order chemical reaction. Furthermore, mRNA is translated in the ribosomes where proteins are the final product. Initiation of translation is considered to be irreversible since the cell utilizes energy in the form of ATP. Kinetic constants for both stages are adjusted so that for each mRNA transcript approximately 20 protein molecules are being produced. Elongation of translation is treated similarly to transcription. Movement of the ribosomal subunits across the mRNA occurs at a rate of 100 AA/sec [41]. Similar to transcription, we model translation as a gamma distributed event [30].

Assumptions that relate to our specific system and have not been mentioned in the previous paragraphs are the following. Monomer forms of TetR protein or fusion of TetR with tranactivators are not able to bind to operator regions. Furthermore we assume that monomer TetR and Tet-OFF are not able to "react" with Tc molecules, only the dimer forms do.

### Model assumptions & initial conditions

The main underlying assumptions on which the model is based are as follows. The reactant volume is considered to be well stirred and the species are diluted in a large number of water molecules (homogeneous). In all simulations we consider a cell which we follow over time as it divides to produce ancestors (cell division with the doubling time of cells being 30 ± 4 min). Each cell is considered to be of initial volume 10^-15 ^liters and then grows exponentially until it divides, with division times following a normal distribution with mean 30 minutes and standard deviation 4 minutes. Furthermore, the species velocities follow a Maxwell Boltzmann distribution leading to a large number of neutral collisions that add to the homogeneity and a small number of reacting collisions. The system is considered to be isolated, that is other genes or organelles are assumed not to interfere, while mass transfer through the "boundaries" is allowed (for example nucleotides bases are readily available). Also, the cell has all the nutrients it needs to fuel its metabolism, which keeps major components (for example, free and available RNA polymerase, proteolytic enzymes) concentrations constant. Temperature and pH are assumed to remain constant throughout the simulations.

Turning our attention to the initial conditions we briefly discuss how they were generated. When inserting a vector in a cell that expresses non natural occurring proteins (proteins that are not expressed naturally within the cell) we do not expect to have any previous accumulation of those proteins. In our case there is no previous accumulation of TetR, TetOFF or TetON and GFP. For TetR this is true because we can use strains of E. coli that do not contain the natural tetracycline resistance operon. TetOFF and TetON are not naturally occurring proteins in any bacterial or mammalian cell and finally GFP is also not naturally expressed in E. coli. Therefore all their concentrations are set initially to zero. This also means that all intermediates involved in their transcription, translation, regulation will also be absent, hence have zero initial concentrations. On the other hand we set the initial concentration of each promoter/operator sites equal to one since that is the amount the cell will recognize, assuming that the plasmid copy number is one (this can be achieved with appropriate choice of plasmid vectors). Finally available and free RNA polymerase and ribosome numbers are set accordingly to represent average values. In our case, all simulations use 180 molecules of free and available RNA polymerase and 300 free and available ribosomes [18,20,42].

#### Hybrid stochastic simulation algorithm

Biological systems, such as gene networks, are far from the thermodynamic limit (small species concentration for example few copies of DNA) therefore a deterministic approach would be invalid. In addition, the observation that biological systems are inherently stochastic [16] allows for an accurate modeling of biomolecular events through discrete – stochastic algorithms. Such algorithms are based on a jump Markov process representation of the system, which accurately captures the fluctuations occurring when systems are far from the thermodynamic limit.

Gillespie's stochastic simulation algorithm (SSA) [43,44] is the first algorithm deployed to exactly simulate the evolution of well-mixed chemical kinetic systems. Improvements in the use of random numbers and introduction of special data structures allowed Gibson and Bruck to create the Next Reaction variant [30]. Still in systems where fast occurring reactions are present, SSA becomes computational expensive, since it simulates each individual reacting event. Many approximations [45,46] have been proposed to overcome the observed slow down. However, the disadvantage is that such approximations suffer when they are not valid for a subset of the system. Therefore a hybrid approach that treats each subset differently using the appropriate mathematical representation, discrete-stochastic, continuous-stochastic or continuous-deterministic, and then merges all the different realizations to produce a fast and accurate solution is a more rigorous approach. Few hybrid methods have been proposed [47-49]. Recently a hybrid algorithm [50] that separates the reactions into two subsets, fast/continuous and slow/discrete has been devised. The first are propagated in time using the Chemical Langevin Equation (CLE) formalism and the later using differential "jump equations". This approach while retaining accuracy, achieves significant efficiencies in the computational time when many fast occurring reaction are present, compared to SSA and other existing hybrid methods [50,51].

Due to the coupling between the system of CLE's and differential "jump equations" they are integrated simultaneously using numerical schemes, such as the Euler-Maruyama or Milstein method which may or may not facilitate the use of adaptive time stepping schemes, depending on the stiffness of the system. A user friendly program that implements the Hybrid Stochastic Algorithm, called Hy3S, is readily available [51]. Hy3S proves to be both accurate and computational inexpensive compared to SSA and its variants when fast/continuous reactions are present, for instance dimerization reactions. In previous work of ours we compare Hy3S with existing hybrid methods and we demonstrate the clear benefits of Hy3S over other existing methods [17,18,20,50,51]. Furthermore there are two additional benefits in using Hy3S: First, Hy3S is publicly available and can be compared with all other algorithms openly. Second, to our knowledge there is no algorithm that has successfully been used in modeling gene regulatory networks at the level of detail we are presenting in this work (all the interactions in transcription, translation, regulation and induction, with kinetic constants spanning 20 orders of magnitude).

We have made available the necessary files for simulating Network IV in the website of Hy3S [52]. Accessibility of the files is plausible through the GUI created for Matlab, which can readily be found in the Hy3S website together with instructions. In these files initial conditions are set as discussed in the 'model assumptions' section in the manuscript and the values used for the kinetic parameters in the case of wild-type dynamics can be observed in Table 1. All files produce 100 independent trajectories and include cell division.

In our work, all realizations were obtained using Itanium2 1.5 GHz processors. Average simulation times for Network IV range from 4 to 6 hours per trial. For example consider the results shown on Figure 6D. The simulation times of the network configurations using the different mutated TetR variants are approximately 4.5 hrs, where the execution times increase slightly as we decrease the binding affinity of TetR for all TetO.

## Competing interests

The author(s) declare that they have no competing interests.

## Authors' contributions

VS carried out the simulations and drafted the manuscript. YK conceived of the study, and participated in its design and coordination and helped to draft the manuscript. All authors read and approve of the final manuscript.

## Figures and Tables

**Table 1:** A Chemical kinetics representation of Network III

**#**	**Reaction**	**k**	**Ref.**	**#**	**Reaction**	**k**	**Ref.**
1	TcEx --> Tc	3.3E-4	[31]	48	TetOFF2 + OP2 --> TetOFF2:OP2	2.86E+06	[34]
2	2 TetR --> TetR2	1.00E+09	[32]	49	TetOFF2:OP2 --> TetOFF2 + OP2	5.11E-04	[34]
3	TetR2 --> 2 TetR	10	[32]	50	TetR2 + OP2 --> TetR2:OP2	2.86E+06	[34]
4	2 TetOFF --> TetOFF2	1.00E+09	[32]	51	TetR2:OP2 --> TetR2 + OP2	5.11E-04	[34]
5	TetOFF2 --> 2 TetOFF	10	[32]	52	TetOFF2:OP2 + Tc --> Tc:2TetOFF:OP2	2.0E+6	[4]
6	Tc + TetR2 -->Tc:2TetR	2.00E+06	[4]	53	Tc:2TetOFF:OP1 + Tc --> Tc:TetOFF2:OP2	1.0E+15	§
7	Tc + Tc:2TetR --> Tc:TetR2	1.0E+15	§	54	Tc:TetOFF2:OP2 --> Tc:TetOFF2 + OP2	5.8E-3	[35]
8	Tc:TetR2 --> TetR2 + 2Tc	0.001	[4]	55	TetR2:OP2 + Tc --> Tc:2TetR:OP2	2.0E+6	[4]
9	Tc + TetOFF2 --> Tc:2TetOFF	2.00E+06	[4]	56	Tc:2TetR:OP2 + Tc --> Tc:TetR2:OP2	1.0E+15	§
10	Tc + Tc:2TetOFF --> Tc:TetOFF2	1.0E+15	§	57	Tc:TetR2:OP2 --> Tc:TetR2 + OP2	5.8E-3	[35]
11	Tc:TetOFF2 --> TetOFF2 + 2Tc	0.001	[4]	58	RNAp + P2 + OP2 --> RNAp:P2:OP2	8.60E+6	[38]
12	TetOFF -->	0.0012	*	59	RNAp:P2:OP2 --> RNAp + P2 + OP2	0.01	[38]
13	TetOFF2 -->	0.0012	*	60	RNAp:P2:OP2 --> RNAp*:P2:OP2	0.013	[38]
14	TetR -->	0.0012	*	61	RNAp*:P2:OP2 --> RNAp*:DNA21 + P2 + OP2	30	[40]
15	TetR2 -->	0.0012	*	62	RNAp + P2 + OP2:TetR2 --> RNAp:P2:OP2:TetR2	8.60E+6	[38]
16	Tc:TetOFF2 --> Tc	0.0012	*	63	RNAp:P2:OP2:TetR2 --> RNAp + P2 + OP2:TetR2	0.01	[38]
17	Tc:TetR2 --> Tc	0.0012	*	64	RNAp:P2:OP2:TetR2 --> RNAp*:P2:OP2:TetR2	0.013	[38]
18	GFP-LAA -->	2.88E-04	[36]	65	RNAp*:P2:OP2:TetR2 --> RNAp*:DNA21 + P2 + OP2:TetR2	30	[40]
19	Tc -->	2.67E-06	[37]	66	RNAp + P2 + OP2:TetOFF2 --> RNAp:P2:OP2:TetOFF2	8.60E+6	[38]
20	TcEx -->	2.67E-06	[37]	67	RNAp:P2:OP2:TetOFF2 --> RNAp + P2 + OP2:TetOFF2	0.01	[38]
21	TetOFF2 + OP1 --> TetOFF2:OP1	2.86E+06	[34]	68	RNAp:P2:OP2:TetOFF2 --> RNAp*:P2:OP2:TetOFF2	0.13	¶
22	TetOFF2:OP1 --> TetOFF2 + OP1	5.11E-04	[34]	69	RNAp*:P2:OP2:TetOFF2 --> RNAp*:DNA21 + P2 + OP2:TetOFF2	30	[40]
23	TetR2 + OP1 --> TetR2:OP1	2.86E+06	[34]	70	RNAp*:DNA21 --> RNAp + mRNA2	30, 621	[40]
24	TetR2:OP1 --> TetR2 + OP1	5.11E-04	[34]	71	mRNA2 -->	0.002	$
25	TetOFF2:OP1 + Tc --> Tc:2TetOFF:OP1	2.0E+6	[4]	72	mRNA2 + Ribosome --> Rib:mRNA2	1.00E+05	$
26	Tc:2TetOFF:OP1 + Tc --> Tc:TetOFF2:OP1	1.0E+15	§	73	Rib:mRNA2 --> Rib:mRNA21 + mRNA2	100	[41]
27	Tc:TetOFF2:OP1 --> Tc:TetOFF2 + OP1	5.8E-3	[35]	74	Rib:mRNA21 --> Ribosome + TetR	100, 207	[41]
28	TetR2:OP1 + Tc --> Tc:2TetR:OP1	2.0E+6	[4]	75	TetOFF2 + OP3 --> TetOFF2:OP3	2.86E+06	[34]
29	Tc:2TetR:OP1 + Tc --> Tc:TetR2:OP1	1.0E+15	§	76	TetOFF2:OP3 --> TetOFF2 + OP3	5.11E-04	[34]
30	Tc:TetR2:OP1 --> Tc:TetR2 + OP1	5.8E-3	[35]	77	TetR2 + OP3 --> TetR2:OP3	2.86E+06	[34]
31	RNAp + P1 + OP1 --> RNAp:P1:OP1	8.60E+06	[38]	78	TetR2:OP3 --> TetR2 + OP3	5.11E-04	[34]
32	RNAp:P1:OP1 --> RNAp + P1 + OP1	0.01	[38]	79	TetOFF2:OP3 + Tc --> Tc:2TetOFF:OP3	2.0E+6	[4]
33	RNAp:P1:OP1 --> RNAp*:P1:OP1	0.013	[38]	80	Tc:2TetOFF:OP1 + Tc --> Tc:TetOFF2:OP3	1.0E+15	§
34	RNAp*:P1:OP1 --> RNAp*:DNA11 + P1 + OP1	30	[40]	81	Tc:TetOFF2:OP3 --> Tc:TetOFF2 + OP3	5.8E-3	[35]
35	RNAp + P1 + OP1:TetR2 --> RNAp:P1:OP1:TetR2	8.60E+6	[38]	82	TetR2:OP3 + Tc --> Tc:2TetR:OP3	2.0E+6	[4]
36	RNAp:P1:OP1:TetR2 --> RNAp + P1 + OP1:TetR2	0.01	[38]	83	Tc:2TetR:OP3 + Tc --> Tc:TetR2:OP3	1.0E+15	§
37	RNAp:P1:OP1:TetR2 --> RNAp*:P1:OP1:TetR2	0.013	[38]	84	Tc:TetR2:OP3 --> Tc:TetR2 + OP3	5.8E-3	[35]
38	RNAp*:P1:OP1:TetR2 --> RNAp*:DNA11 + P1 + OP1:TetR2	30	[40]	85	RNAp + P3 + OP3 --> RNAp:P3:OP3	8.60E+6	[38]
39	RNAp + P1 + OP1:TetOFF2 --> RNAp:P1:OP1:TetOFF2	8.60E+6	[38]	86	RNAp:P3:OP3 --> RNAp + P3 + OP3	0.01	[38]
40	RNAp:P1:OP1:TetOFF2 --> RNAp + P1 + OP1:TetOFF2	0.01	[38]	87	RNAp:P3:OP3 --> RNAp*:P3:OP3	0.013	[38]
41	RNAp:P1:OP1:TetOFF2 --> RNAp*:P1:OP1:TetOFF2	0.13	¶	88	RNAp*:P3:OP3 --> RNAp*:DNA31 + P3 + OP3	30	[40]
42	RNAp*:P1:OP1:TetOFF2 --> RNAp*:DNA11 + P1 + OP1:TetOFF2	30	[40]	89	RNAp*:DNA31 --> RNAp + mRNA3	30, 723	[40]
43	RNAp*:DNA11 --> RNAp + mRNA1	30, 660	[40]	90	mRNA3 -->	0.002	$
44	mRNA1 -->	0.002	$	91	mRNA3 + Ribosome --> Rib:mRNA3	1.00E+05	$
45	mRNA1 + Ribosome --> Rib:mRNA1	1.00E+05	$	92	Rib:mRNA3 --> Rib:mRNA31 + mRNA3	100	[41]
46	Rib:mRNA1 --> Rib:mRNA11 + mRNA1	100	[41]	93	Rib:mRNA31 --> Ribosome + GFP-LAA	100, 248	[41]
47	Rib:mRNA11 --> Ribosome + TetOFF	100, 220	[41]				

Reactions and kinetic rates for the chemical kinetics representation of network III. Units on k: 1^st ^order reaction: sec^-1^, 2^nd ^order: (M sec)^-1^, power law kinetics M^-2 ^sec^-1^. Reactions with two kinetic constants are γ - distributed events, where the first number is the rate of each step and the second is the number of steps. References are noted next to each reaction. §: rate adjusted for fast reacting intermediates. *: rates adjusted for 10 min half-life. ¶: rate adjusted from the function of activator. $: rates adjusted to give 20 protein molecules per mRNA transcript. Numbering of the genes starts from top to bottom as they are shown in Figure 1. For example, P1: promoter of gene 1, OP1: operator of gene 1.
